# Digging into the roots: understanding direct and indirect drivers of ecosystem service trade-offs in coastal grasslands via plant functional traits

**DOI:** 10.1007/s10661-020-08817-x

**Published:** 2021-05-14

**Authors:** Miguel A. Cebrián-Piqueras, Juliane Trinogga, Anastasia Trenkamp, Vanessa Minden, Martin Maier, Jasmin Mantilla-Contreras

**Affiliations:** 1 Department of Agricultural Economics and Rural Development, University of Göttingen, Platz der Göttinger Sieben 5, 37073 Göttingen, Germany; 2 Institute of Biology and Environmental Sciences, University of Oldenburg, Carl von Ossietzky Str. 9-11, D-26129 Oldenburg, Germany; 3 Ecology and Environmental Education Group, Institute of Biology and Chemistry, University of Hildesheim, Universitätsplatz 1, 31141 Hildesheim, Germany; 4 Department of Biology, Ecology and Biodiversity, Vrije Universiteit Brussel, 1050 Brussels, Belgium

**Keywords:** Trait-based approach, Functional ecology, Partial least squares structural equation modeling (PLS-SEM), Biodiversity effects, Land use intensification, Grassland communities, Plant-soil interactions, Leaf economics spectrum, Root traits

## Abstract

Recent empirical and theoretical approaches have called for an understanding of the processes underpinning ecosystem service provision. Environmental gradients have shown effects on key plant functional traits that subsequently explain ecosystem properties of several systems. However, little is known concerning how associations between plant functional traits, including both below- and aboveground plant components, predict ecosystem properties and independently measured final ecosystem services. Here, we modeled (1) the responses of the leaf and plant economics spectrum, Plant size axis, and root growth to environmental gradients and (2) how associations between plant functional traits explain trade-offs and synergies between multiple ecosystem properties and final services. Forty-four plots were studied in a coastal marsh landscape of the German North Sea Coast. We used a partial least square structural equation model approach to test the hypothesized model. We found (1) a negative covariation between plant traits pertaining to a size axis and traits explaining both plant growth (roots and stems) and the leaf economics spectrum; (2) this trade-off responded significantly to the land use gradient and nutrient availability, which were both strongly driven by the groundwater gradient; (3) this trade-off explained an initial major trade-off between carbon stocks, at one extreme of the axis, and both the habitat value to conserve endangered plants and forage production for meat and dairy products at the other extreme. However, a secondary trade-off between nature conservation value and forage production, explained by a trade-off between leaf economics spectrum and plant growth in response to the land use intensity gradient, was also found.

**Supplementary Information:**

The online version contains supplementary material available at 10.1007/s10661-020-08817-x.

## Introduction

Ecosystem services (ES) are the benefits that humans obtain from ecosystems. For achieving some of these benefits, strong landscape modifications are deliberately exerted (MEA, 2005). For instance, in agroecosystems, maximizing the procurement of provisioning services, i.e., products directly obtained from ecosystems, e.g., food, fiber, or timber, has frequently weakened the ecosystems’ capacity to provide other supporting services (i.e., basis for the services of the other categories, e.g., nutrient cycling, biomass production), regulating services (i.e., benefits obtained from the regulation of ecosystem processes, e.g., water, soil, or climate regulation), and cultural services (i.e., non-material benefits people obtain from nature, e.g., recreation or esthetic value of ecosystems) (Bennett & Balvanera 2007; Power 2010). Negative relationships between the ES have been called ES trade-offs. ES trade-offs occur when maximizing the procurement of one given ES hampers the capacity of the ecosystems to supply another ES (Raudsepp-Hearne et al*.* 2010). In contrast, ES synergies arise when the use of one ES directly increases the benefits supplied by another service. Research approaches using land cover data as proxies for the ES supply are increasingly being used to identify spatial ES trade-offs and co-occurrence of ES, namely ES bundles, i.e., ES bundles have been defined as sets of ecosystem services that repeatedly appear together across space or time (Raudsepp-Hearne et al. 2010). Land cover approaches using proxies can be suitable for identifying broad-scale trends in ES supply, but even relatively good proxies are likely to be unsuitable for identifying hotspots or priority areas for multiple services, as they can fail to accurately predict the mechanistic understanding of ES supply since the biophysical processes underpinning ES are usually not accounted (Eigenbrod et al. 2010, Bennett et al. 2009). Mechanistic and process-based approaches are therefore emerging as promising tools to understand and predict changes in the provision of biodiversity and multiple ecosystem services and assess the roots of ecosystem service trade-offs (Bennett et al. 2009, Duncan et al. 2015). For example, some studies in mountain grasslands have quantified significant direct effects of climate and land use on multiple ES via plant functional traits at spatial scale (Lamarque et al. 2014). Understanding of process-based relationships e.g. responses of biodiversity to drivers and its effect on multitude of ecosystem services, appears to be aprerequisite for a proper understanding of spatial distribution of ES and their trade-offs as shown by previous works of Lavorel et al. (2011) or Lamarque et al. (2014). Specifically, within ecosystems, vegetation plays a central role on explaining a bright spectrum of not only regulating, provisioning, and supporting but also cultural ES (De Bello et al. 2010). Vegetation is an essential target of research addressing the connection between biodiversity-ecosystem functioning-ecosystem services (B-EF-ES) (Isbell et al. 2011, Duncan et al. 2015), and it is key for the identification of the relationships between ES and several trophic levels (Lavorel et al. 2013).

Trait-base approaches have extensively been used to predict changes of vegetation, via plant functional traits, in response to climate and land use to explain ecosystem functions (Minden & Kleyer 2015, Conti & Díaz 2013). As defined by Violle et al. (2007) “functional traits are morpho-physio-phenological traits which impact fitness indirectly via their effects on growth, reproduction and survival, the three components of individual performance”. Strong evidence of plant functional trait associations contributing to fundamental ecosystem functions has been found, especially for plant trait effects on primary production and some processes associated with carbon and nitrogen cycling (Lavorel 2013). Besides, trait-based approaches are increasingly been used to explain multiple vegetation-mediated ES (Hevia et al. 2017) and their trade-offs (Lavorel & Grigulis 2012). However, understanding these processes requires a profound knowledge of the complex associations and coordination between plant functional traits in response to environmental factors and land use (Lavorel & Grigulis 2012, Wen et al. 2019).

Plant functional traits vary in response to environmental gradients (Lavorel & Garnier 2002; Paula & Pausas 2011). Variation of key leaf functional traits (e.g., specific leaf area (SLA), leaf nitrogen content (LNC), and leaf dry matter content (LDMC)) has been found to indicate a trade-off between a resource acquisition strategy (i.e., high values of SLA and LNC and low values of LDMC) and a resource conservation strategy (i.e., high values of LDMC and low values of SLA and LNC). This trade-off has globally been proven showing wide-ranging and convincing evidence that feasible leaf investment strategies are to a great extent arrayed along a single spectrum, namely leaf economics spectrum (LES, Wright et al. 2004). Variation of these functional traits strongly responds to biomass removal (Lienin & Kleyer 2011), soil nutrient availability, or elevation gradients (Lavorel & Grigulis 2012). Yet, associations between leaf economics spectrum traits and other plant traits, e.g., belowground traits, are poorly understood and need to be addressed (Kleyer et al. 2018). Evidence of a coordinated whole plant economics spectrum as an extension of the leaf economics spectrum was described (Freschet et al. 2010), showing that variation in root, stem, and leaf traits operate in a coordinated fashion in response to environmental changes. However, research on this direction needs to consider different environmental gradients, scales, and ecosystems to draw accurate conclusions for specific contexts (Wood et al. 2015, Hevia et al. 2017). Studies on a global scale have shown an alternative group of plant functional traits that may operate independently from the leaf economics spectrum (Díaz et al. 2004). Plant height or biomass dry weights are key plant traits associated with the so-called size axis (Westoby et al. 2002). Plant traits explaining the size axis are good candidates to predict plants’ abilities to capture light resources (Westoby et al. 2002), plant life history (Moles & Westoby 2006), or reproductive abilities (Grime 1977; Westoby & Wright 2006). A co-variation between size axis and leaf economics spectrum in response to biomass removal was found in agricultural systems from central Europe. However, when several systems were included, e.g., both managed grasslands and heaths, this relationship was not obvious (Lienin & Kleyer 2011).

Recently, the responses of several plant functional traits to environmental parameters such as saline groundwater have been studied in temperate saltmarshes (Minden and Kleyer 2011 and 2015). However, studies showing response effects on broader scales in such temperate coastal grasslands, including both freshwater and saltwater systems, has not yet been addressed. The “biomass ratio” hypothesis (Grime 1998) states that dominant species from a community control the main ecosystem properties (EP); conversely, less abundant species do not exert significant effects on these properties, i.e., by contrast, some authors have shown that the functional diversity of plant traits (e.g., functional richness, evenness, or divergence) controls key ecosystem functions due to the complementarity in using resources, i.e., “Niche complementary or Diversity Hypothesis” (Wen et al. 2019, Díaz et al. 2007, Tilman 1997). However, models associated with the two hypotheses are not necessarily mutually exclusive as both have been shown to operate in natural ecosystems and can have different relative importance in different situations (Conti & Díaz 2013). Following the biomass ratio hypothesis, recent empirical studies have shown evidence of how plant functional traits measured at the community-level respond to environmental gradients by using community-weighted means of traits. Trade-offs and associations between these plant functional traits drive, subsequently, trade-offs and synergies between ecosystem properties (Minden & Kleyer 2015). These coordinated and structured relationships between functional traits and ecosystem properties explain single or multiple-service interactions (Conti & Díaz 2013; Lavorel & Grigulis 2012, Hevia et al. 2017). For example, biomass production is associated with several plant functional traits (e.g., canopy height, root or stem biomass) (DeBello et al. 2010; Minden and Kleyer 2011), and it is related to ecosystem services such as carbon sequestration (Conti & Díaz 2013), yield production (Lavorel et al. 2011), or wave attenuation (Maza et al 2015). Still, most of the research has so far focused either on the effects on single services instead of multiple services, including bundles, or on pure biophysical properties instead of final ecosystem services (Wong et al. 2015; Duncan et al. 2015).

Here, we asked how variations in community-weighted mean values of plant functional traits related to leaf/plant economics spectrums and size axis, in response to land use and environmental parameters, explain trade-offs and synergies between ecosystem carbon stocks (regulating service) and final values of ecosystem services such as the habitat value to conserve endangered plants (existence and intrinsic values and cultural service) (MEA 2005) and forage production for meat and dairy products (provisioning service) (MEA 2005) in temperate coastal grasslands. We tested our approach in a coastal grassland system of North-West Germany (44 plots). Associations between soil organic carbon and aboveground biomass stocks are being increasingly studied (Conti & Díaz 2013; Doblas Miranda et al. 2013), yet the relationship between ecosystem carbon stocks and final services is poorly understood (Lamarque et al. 2014). We propose a functional trait-based approach that uses a structural equation model formalism (Lavorel & Grigulis 2012) based on the effect-response framework (Lavorel & Garnier 2002; Minden & Kleyer 2011) to test our hypothesized initial model (Fig. 2). Here, we extend the effect-response approach by incorporating both supporting services (ecosystem properties that support the provision of final services) and values of final services, understood as components of nature having an effect on human well-being and direct value to society, following Wong et al. (2015) and Duncan et al. (2015).

Specifically, our goals were:

1. To identify trade-offs and associations between plant functional traits in a particular coastal grassland system;
2. To quantify responses of plant functional traits to abiotic and land use parameters;
3. To disentangle how associations and trade-offs between plant functional traits explain ecosystem properties and ES trade-offs and synergies in relation to (i) forage production for meat and dairy products, (ii) habitat value to conserve endangered plants, and (iii) carbon stocks.

## Methods

Below, we carry out an exhaustive description of (1) the study site; (2) the data collection of abiotic ecosystem properties, land use, plant traits, biotic ecosystem properties, and indicators of final ecosystem services; and (3) the data analysis using a path modeling, specifically partial least squares structural equation model (PLS-SEM). Finally, (4) we present the hypothesized initial model containing the expected associations and cause-effect relationships between parameters based on previous works.

### Site description

**Fig. 1:**
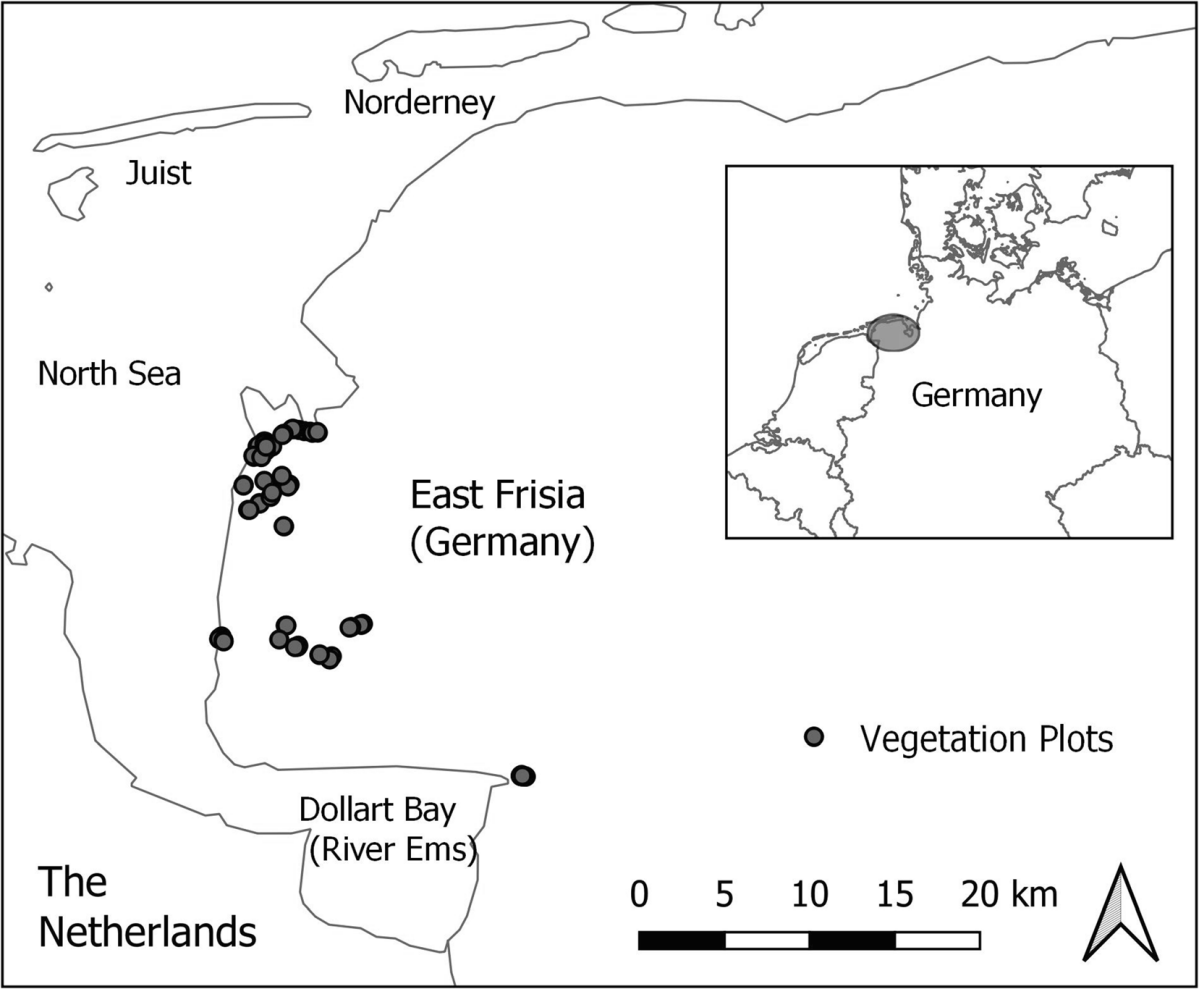
The study site was located on East Frisia, North-West coast of Germany. The plots were distributed within the communities of Krummhörn and Emden covering main grassland vegetation types: salt marsh, reeds, wet and mesic pastures. Grey-colored points indicate the plots where the abiotic, biotic, and ecosystem service data was measured (*N*, 44)

The study site was located in a temperate coastal marsh landscape of East Frisia (E 07° 02′, N 53° 27′, NW-Germany). It has a mean annual temperature of 9.4 °C, an elevation ranging from − 2.5 to 1.5 m above sea level, and a mean of 823 mm annual rainfall (German meteorogical service, 2018). Forty-four plots (4 m^2^) were randomly selected from four major grassland ecosystems, ranging from (i) low saltmarshes (*Puccinellia maritima*, *Atriplex prostrata*, *Aster tripolium*, *Suaeda maritima*) and high saltmarshes (*Elymus athericus*, *Aster tripolium*, *Artemisia maritima*); (ii) reeds (*Phragmites australis*); (iii) wet extensive grasslands (*Festuca rubra*, *Juncus gerardi*, *Elymus repens*, *Potentilla anserina*, *Cynosurus cristatus*); and (iv) intensively, fertilized mesic grasslands (e.g., *Lolium perenne*, *Dactylis glomerata*, *Agrostis stolonifera*) (Fig. 1) following previous works in similar systems (Minden and Kleyer 2011). Data was collected in this site between 2011 and 2014.

### Measurements of abiotic ecosystem properties (abiotic EP)

For each soil horizon of each plot down to a depth of 80 cm, soil samples were collected in March 2012 with a soil-sample ring of 100 cm^3^ and were then air dried and sieved. Bulk density was evaluated from 200 cm^3^ of soil (Schlichting et al. 1995). From each plot, plant-available potassium (K) and phosphorus (P) were extracted with ammonium lactate-acetic acid at pH 3 (Egnér et al. 1960) and analyzed using AAS (atomic absorption spectroscopy) and CFA (continuous flow analyses) (Murphy & Riley 1962), respectively. Calcium carbonate (CaCO_3_) was extracted following Scheibler, in Schlichting et al. (1995).

At each plot, a drainage pipe (10-cm diameter) was vertically installed to a depth of 80 cm in the ground. In these pipes, mean groundwater levels were recorded biweekly during the vegetation period between March and October 2012 following Minden and Kleyer (2011). Additionally, for plots in which variation in water levels was common, i.e., reeds, salt marshes, and wet meadows, groundwater was data-logged every half an hour with Sensus Ultra Divers (Reefnet Inc.) between May and October 2012. Along with the fortnightly groundwater recordings, groundwater electrical conductivity was measured with WTW ph/Cond340i/SET, using a Tetracon 325 electrode as a proxy for salinity.

### Measurements of land use

Grassland fields were grazed, mown, or both with varied intensity (17:44 plots were grazed by cattle (M 7.8; SD 16.0; Max. 50, Min. 1 (livestock units/ha)) and 10:44 plots were mown (M 2.2; SD 1.3 Max. 4.5, Min. 1 (times a year). Five plots were both grazed and mown. Biomass removal was quantified using fenced exclosures of 4 m^2^, where grazing or mowing was prevented. Aboveground biomass was then sampled at 50-mm stubble height both inside and outside the fence on each plot from randomly selected 1-m^2^ subplots in March and August, representing the beginning and peak biomass periods (de Leeuw et al. 1990). Samples were sorted according to live and dead biomass, oven dried at 70 °C for 72 h, and then weighed. The influence of management in percent was calculated by dividing the values inside and outside the exclosures, times 100. The parameter “biomass removal” was calculated as the sum of (i) the herbage consumed by the cattle and biomass removed by mowers and (ii) losses of herbage due to cattle trampling or haymaking, as a percentage of maximum standing biomass at each plot. Biomass values given refer to 1m^2^. Aboveground standing biomass (g m^−2^): mean 1329.20, SD 723.84.

### Measurements of plant traits

Following previous standardized methodologies (Garnier et al. 2007) (see Supplementary Material (SM) 1 for a comprehensive description), individual plants from which plant traits’ measurements were taken were collected from 304 locations in 4 regions (Western Pommerania, DE; Aarhus, DK; Zeeland, NL; East Frisia, DE) (Table 1 and SM 2) covering main coastal grassland vegetation types (coastal marshes, extensive wet grasslands, mesic pastures, and reeds). Plant trait measurements were collected between 2006 and 2014. Plant species composition and abundance were recorded by frequency analysis at each plot of the study site (44 plots) in the summer of 2012, using a 1 × 1 m grid of 100 cells (each 10 × 10cm) following Tremp (2005). Frequency analysis was conducted within exclosures for grazed or mowed plots.

**Table 1:** Description of the 4 regions where trait plants were collected

Location	Lat.	Lon.	MAT (°C)	MAR (mm)	Number of plots	Main plant communities
Western Pommerania, DE	54° 20′	12° 42′	8.2	553	48	Fens, wet and mesic pastures
Aarhus, DK	56° 10′	10° 41′	7.8	605	44	Salt marsh, dry coastal grasslands, wet and mesic pastures
Zeeland, NL	51° 28′	3° 41′	10.1	733	39	Salt marsh, reeds, wet and mesic pastures
East Frisia, DE	53° 24′	7° 06′	8.8	786	173	Salt marsh, reeds, wet and mesic pastures

*Lat* latitude, *Lon* longitude, *MAT* mean annual temperature, *MAR* mean annual rainfall, and main plant communities

A total number of 2104 plant individuals (92 different plant species) were collected during 2006–2014 from the 304 plots. The trait plants were collected in the abovementioned four regions. We selected the most abundant herbaceous species that collectively added up to approx. 80% of the plot biomass (Garnier et al. 2007). Morphological traits such as canopy height were directly measured in the field. Other morphological traits (i.e., specific leaf area), chemical traits (i.e., leaf nitrogen content), and biomass traits (i.e., leaf dry weight) were analyzed and calculated in the laboratory of the University of Oldenburg (see methods below). From 1318 individual plants, soil blocks (20 × 20 × 40 cm), including roots, were collected in the field to measure root traits. Roots and rhizomes were cleaned and separated with tweezers. Plant organs were separated, oven dried at 70 °C for 72 h, and weighed. We scaled-up from species plant traits to the community level following the “biomass ratio” hypothesis (Grime 1998). Mean trait values of every species were weighted by individual species’ frequency per plot and subsequently averaged by the total frequency of all the species occurring on each plot from the study site (44 plots, see Fig. 1). Thus, the community-weighted mean (CWM) trait values for each plot were obtained (Violle et al. 2007). Seed number per plant individuals were measured by direct counting and extrapolation method (Kleyer 2008).

Specific lengths of stems and roots were calculated as the ratio between the length of the organ section and its dry weight. SLA (specific leaf area) was calculated as the ratio of leaf area to leaf dry mass (mm^2^ mg^−1^) following Pérez-Harguindeguy (2013). Dry matter content of stems, roots, and leaves was measured as the dry mass of the organ section divided by its water-saturated fresh mass (mg^−1^ g^−1^), following Kleyer et al. (2008).

For each plant organ (leaves, stems, roots, and rhizomes), nitrogen and carbon content were analyzed using CHNS-Analyzer Flash AE (Thermo Electron Corp., DE) following Grimshaw et al. (1989). Nitrogen content was measured after grinding the plant material in a planetary mill for 2 to 10 min at 300 to 400 revolutions (pulverisette 7, Fritsch). Each sample was then dried at 105 °C for 4 to 5 h. Two to three milligrams of material was placed in tin tubes (0.1 mg precision balance CP 225 D, Sartorius; tin capsules for solids, Säntis Analytical) and analyzed using the abovementioned analyzer.

### Measurements for biotic EP

Soil organic carbon was determined for the first 80 cm as the difference between CaCO_3_ and total carbon values. For this study, we used soil organic carbon (hereafter SOC) down to a depth of 30 cm because of the expected greater influence from current vegetation and land use. Total soil carbon was measured in all horizons for the upper 80 cm of soil.

The aboveground standing biomass was collected in August 2012 (within exclosures for grazed and/or mowed plots). Biomass samples were oven dried at 70 °C for 72 h. Biomass values correspond to 1 m^2^.

Plant species richness per plot (hereafter “plants”) was determined from the frequency analysis (see above section).

Plot species-based forage values were obtained by community-weighed means of the forage value of all vascular plant species of a plot (“Futterwertzahlen” (FW), Briemle et al. 2002). These values indicate the forage value of plant species on an ordinal scale from 1 to 9 based on several parameters, e.g., protein and mineral content, palatability for livestock, or proportion of valuable plant parts (leaves, stems, flowers, fruits). The forage value of plant species was retrieved from the BIOLFLOR database (Klotz et al. 2002).

Litter mass loss (or decomposition rate) was determined using a litterbag experiment. Fresh plant material was collected in the autumn of 2011 and left to decompose for 12 months in the field on the soil surface in 1-mm mesh litterbags (5 g per litterbag and six replicates per plot). The recovered material was oven dried at 70 °C for 72 h and weighed. The rate of litter mass loss was calculated relative to its initial mass as the rate of biomass decomposition per day (%/day) (Garnier et al. 2007).

### Ecosystem service values

The habitat value to conserve endangered plant species (endangered plants) was calculated as an index based on the endangerment category of the plant species occurring at plot level (www.floraweb.de). Plant species were assigned an ordinal value based on the German Red List (für Naturschutz 2011) translated into IUCN endangerment categories. Not evaluated (NE) and lesser concern (LC), 0; near threatened (NT), 1; vulnerable (VU), 2; endangered (EN), 3; critically endangered (CR), 4. Subsequently, the final plot value for the endangered plant species’ variable was the sum of all species’ values from the species that occurred in a plot.

Sales of forage-based products, namely meat and dairy products (forage sales), were obtained from face-to-face interviews with farmers providing information about the gross economic benefit obtained from the land management per hectare and year in € during 2012. Permits for setting the study plots were previously obtained from farmers. Afterwards, farmers involved in the study (*n*, 9) accessed to answer a set of questions in relation to (1) land use (e.g., grazing intensity, mowing intensity, and fertilization rates) and (2) economic benefits for the year 2012.

### Statistical analysis

**Fig. 2:**
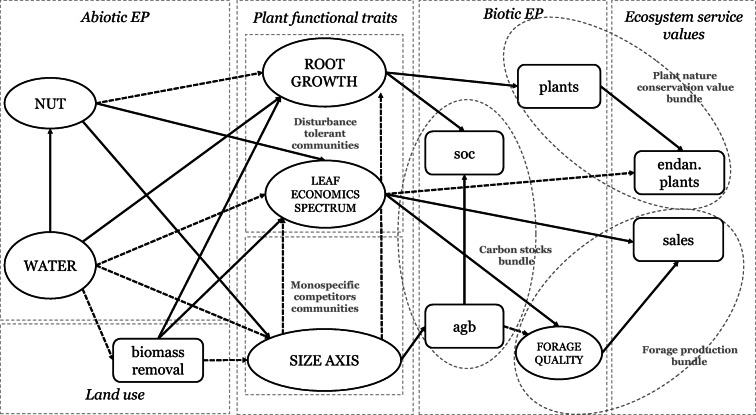
Initial model with hypothesized expected pathways and latent variables. Dashed arrows represent expected negative effects, and solid arrows represent expected positive effects. Latent variables are indicated with ovals and caps lock. Abbreviations and definitions: EP, ecosystem properties; NUT, positive association of plant-available soil nutrients (i.e., nitrogen, potassium, and phosphorous); WATER, positive association of groundwater level and salinity; biomass removal, extraction of biomass due to animal grazing and mowing. ROOT GROWTH, positive association of community-weighted mean (CWM) of specific root length (SRL) and root nitrogen content (RNC) with a negative association with root dry matter content (RDMC); LEAF ECONOMICS SPECTRUM, positive association of CWM of specific leaf area (SLA) and leaf nitrogen content (LNC) with negative association with CWM of leaf dry matter content (LDMC); SIZE AXIS, positive association of CWM of canopy height, leaf dry weight, stem dry weight, leaf area, and seed number; soc, soil organic carbon; agb, aboveground standing biomass; plants, plant species richness; FORAGE QUALITY, positive association of community-weighted mean of species-based forage quality and vegetation decomposition rates; endan. plants, habitat value to conserve endangered plant species; sales, sales from forage-based products, namely meat and dairy products

The initial model (Fig. 2) was tested using PLS-SEM. We used the software Smart-PLS V2.0 (Ringle et al. 2005). Contrary to covariance-based structural equation models (CB-SEM), which have since long been used in ecological research (Grace & Keeley 2006), PLS-SEMs have been extensively used in social science research (Hair et al. 2011; Lowry & Gaskin 2014) and only recently introduced into ecological studies (Peppler-Lisbach et al. 2015; Cebrián-Piqueras et al. 2017a) and social-ecological research (Cebrián-Piqueras et al. 2017b). PLS-SEM enables researchers to estimate complex models with many constructs, indicator variables, and structural paths without imposing distributional assumptions on the data and relatively low sample size. This is possible because PLS-SEM algorithm computes partial regression relationships in the measurement and structural models by using separate ordinary least squares regressions (Hair et al. 2011). Additionally, one of the strengths of PLS-SEM is the use of latent variables. Latent variables are “non-observed” variables inferred from several indicator variables, i.e., “observed” variables (Grace & Keeley 2006). Latent variables usually represent concepts, constructs, or strategies, which are not easily measured directly, though can be explained by multiple factors. For practical reasons is not possible to measure all the factors explaining latent concepts, however, selection of key indicators and the use of PLS-SEMs have been proven to help elicit relevant latent constructs in vegetation ecology, e.g., soil and structural heterogeneity (Peppler-Lisbach et al. 2015; Grace & Keely 2006), or social-ecological research, e.g., social representations of landscapes and ecosystem service provision (Cebrián-Piqueras et al. 2017 b). For instance, we used here hypothesized latent constructs to reduce complexity by using (1) multiple correlated plant functional traits explaining plant strategies and (2) multiple correlated environmental factors explaining gradients. Finally, when the research objectives make more emphasis on exploration than theory confirmation, PLS-SEM is preferred (Hair et al. 2011).

**Fig. 3:**
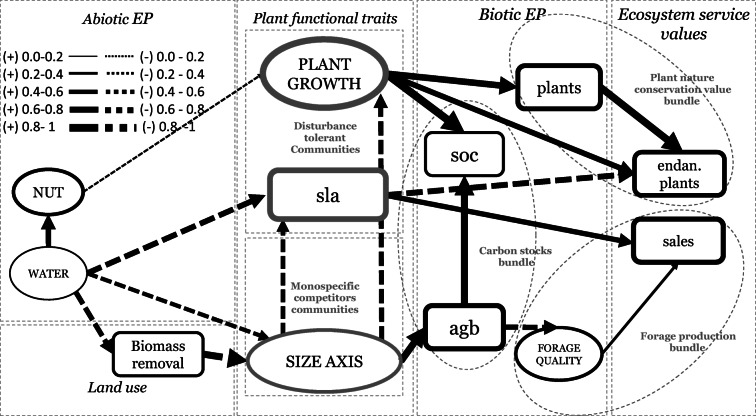
Final model results. Path arrows represent PLS-SEM regression beta path coefficient values. Dashed arrows represent negative effects. Solid arrows represent positive effects. The explained *R*^2^ for endogenous variables is indicated by the frame thickness (see legend inset). Abbreviations and definitions: EP, ecosystem properties; NUT, positive association of plant available soil nutrients (i.e., nitrogen, potassium, and phosphorous); WATER, positive association of groundwater level and salinity; biomass removal, extraction of biomass due to animal grazing and mowing. PLANT GROWTH, positive co-variation of community-weighted mean (CWM) of specific root length (SRL) and specific stem length (SSL); sla, CWM of specific leaf area; SIZE AXIS, positive association of CWM of canopy height, leaf dry weight, stem dry weight, leaf area, and seed number; soc, soil organic carbon; agb, aboveground standing biomass; plants, plant species richness; FORAGE QUALITY, positive association of community-weighted mean species-based grassland forage value and vegetation decomposition rates; endan. plants, habitat value to conserve endangered plants; sales, sales from forage-based products, namely meat and dairy products

Several variables, i.e., habitat value to conserve endangered plants and sales of forage-based products, showed a high heterogeneity and many zero values (i.e., non-normal distribution). Therefore, we decided to use a partial-least squares SEM, instead of a covariance-based SEM, due to the difficulties in normalizing the parameters (Hair et al. 2011). A bootstrap analysis of 5000 runs was used to test path significance. All paths showing bootstrapped path values lower than 1.95 (significance level 5%) were removed from the model (see differences between Figs. 2 and 3) because they were not significant (Hair et al., 2011). Following Hair et al. (2011), we used several measures to check model quality: (1) model internal consistency reliability, composite reliability should be higher than 0.70 (in exploratory research, 0.60 to 0.70 is considered acceptable); (2) indicator reliability, indicator loadings should be higher than 0.70; (3) convergent validity, the average variance extracted (AVE) should be higher than 0.50. Expected latent variables, which were not consistent due to low indicator loadings or low average variance, were modified by removing non-significant indicator variables following (Hair et al. 2011)

### Initial model

Based on prior knowledge, Fig. 2 summarizes the hypothesized initial model, with the expected response effects and latent variables. We hypothesized several latent variables as indicated in Fig. 2. Table 2 shows the expected indicator variable associations explaining latent variables. A positive association of plant-available soil nutrients, such as phosphorus, potassium, and nitrogen, was expected as shown in similar coastal systems in respond to soil texture (i.e., sand, silt, and clay) (Minden & Kleyer 2011). Clayed soils, having higher cation-exchange capacity, are proven to retain better soil nutrients. This latent variable was called soil nutrient availability (hereafter NUTRIENTS). We also hypothesized a positive co-variation of groundwater level and salinity (Minden & Kleyer 2011; Cebrián-Piqueras et al. 2017a) (i.e., closer plots to coast line with higher groundwater level and salinity and more distance plots with lower groundwater level and fresher water). This latent variable was called water gradient, hereafter WATER. A positive association between community aboveground biomass decomposition and the community species-based forage value was expected as, for instance, plants with higher decomposition rates are associated with less lignin content and higher nitrogen content (e.g., leaf nitrogen content) (White et al. 2004; Fortunel et al. 2009; Bakker et al. 2011). Many factors are usually associated with forage quality (i.e., nutrients, energy, protein, digestibility, fiber, or palatability). Here, in order to reduce complexity, we used these two indicator variables, already accounting for most variables associated with forage quality, to express the value of vegetation for cattle forage. This latent variable was called FORAGE QUALITY.

**Table 2:** Parameters used in the model (*N* 44): (1) abiotic ecosystem properties, (2) land use parameters, (3) plant traits, (4) biotic ecosystem properties, and (5) final ecosystem services

Latent variables	Indicator variables/observed variables	Abbreviation	Expected association	Unit	Mean/Median	SD	Max.	Min.
(1) Abiotic ecosystem properties								
Nutrients	Plant available soil potassium content	K	+	g m^−2^ (80 cm. depth)	429.30	229.16	837.79	9.57
	Plant available soil phosphorous content	P	+	g m^−2^ (80 cm. depth)	48.71	26.62	134.55	5.66
	Plant available soil nitrogen	N	+	g m^−2^ (30 cm. depth)	5.46	3.53	15.71	0.98
Water	Mean groundwater level	GW	+	cm	−42.79	22.51	−7.66	−100.00
	Mean groundwater conductivity	Salinity	+	mS cm^−1^	13.65	11.14	37.32	0.00
(2) Land use								
	Biomass removal			% m^−1^	31.07	36.59	91.94	0.00
(3) Plant traits								
Root growth	CWM specific root length (SRL)	SRL	+	mm mg^−1^	32.09	18.32	70.39	7.36
	CWM root nitrogen content (RNC)	RNC	+	%	1.11	0.11	1.44	0.95
	CWM root dry matter content (RDMC)	RDMC	-	mg g^−1^	228.59	44.57	329.13	159.29
Stem growth	CWM specific stem length (SSL)	SSL	+	mm mg^−1^	2.79	2.08	8.69	0.36
	CWM stem nitrogen content (SNC)	SNC	+	%	0.73	0.14	1.17	0.54
	CWM stem dry matter content (SDMC)	SDMC	-	mg g^−1^	293.10	69.92	452.42	172.51
Leaf economics spectrum (LES)	CWM specific leaf area (SLA)	SLA	+	mm^2^ mg^−1^	20.86	5.95	32.06	13.42
	CWM leaf nitrogen content (LNC)	LNC	+	%	1.80	0.38	3.01	1.13
	CWM leaf dry matter content (LDMC)	LDMC	-	mg g^−1^	324.30	71.94	440.73	220.52
Size axis	CWM canopy height (CH)	CH	+	g	63.44	46.33	150.46	12.14
	CWM stem dry weight (SDW)	SDW	+	g	2961.45	2968.39	8637.03	146.66
	CWM leaf dry weight (LDW)	LDW	+	g	959.30	952.32	2985.90	85.36
	CWM leaf area (LA)	LA	+	mm^2^	1513.95	1470.97	4615.38	100.88
	CWM seed number	SN	+	n	1535.53	2233.97	6081.23	104.93
(4) Biotic ecosystem properties								
*	Plant species richness	Plants		n	7	3.69	15	1
*	Soil organic carbon	SOC		Kg m^−2^ (30-cm depth)	10.58	4.19	20.31	2.01
*	Above-ground standing biomass	AGB		g m^-2^	1329.20	723.84	071.60	229.90
Forage quality	Forage value (CWM)	Forage value	+	Index	6	1.80	9	3
	Native biomass decomposition rate	Decomposition	+	%/day	0.26	0.10	0.45	0.01
(5) Final ecosystem services								
*	Habitat value to conserve endangered plants	Endan. plants		Index	0	2.05	8	0
*	Sales of forage-based agricultural products	Sales		€/ha/year	931.59	1380.31	4400	0

The table summarizes the expected latent variables and indicator variables. Latent variables are the theoretical or conceptual elements in the structural model (unobserved variables). Latent variables are explained by indicator variables (observed variables)

*Single observed variables which are not expected to explain latent variables. The expected positive and negative association of indicator variables is expressed with (+) and (-) symbols respectively

*CWM* community-weighted mean

#### Plant functional trait associations and trade-offs

Following previous works confirming the existence of root economics spectrum (i.e., resource conservation vs. resource acquisition) (Paula & Pausas 2011; Prieto et al. 2015), we expected a positive association of plant traits’ indicators of a soil resource acquisition strategy such as fine root growth, i.e., root nitrogen content (RNC), specific root length (SRL), and a negative correlation with root dry matter content (RDMC), associated with conservation of resources. We called this latent variable ROOT GROWTH

In a similar fashion, and following the “leaf economics spectrum” indicating a trade-off between conservation of resources and acquisition explained by leaf plant traits (Wright et al. 2004), a positive association between leaf traits such as leaf nitrogen content (LNC) and specific leaf area (SLA), with a negative association to leaf dry matter content (LDMC), was also expected. We called this latent variable leaf economics spectrum (LES).

A positive co-variation of aboveground biomass dry weight such as stem and leaf with canopy height (CH) and leaf area (LA) was expected based on global results confirming a recurrent size-related axis where taller plants, with higher biomass, are displaying bigger leaves and heavier seeds (Lavorel and Grigulis 2012, Díaz et al. 2004). We also expected an association of reproductive abilities, indicated by seed number, and the size axis, following competitors vs. ruderals trade-off in a highly productive environment (Grime 1977). This latent value was called SIZE AXIS.

##### Traits’ responses to the environment

We expected that the variation of environmental parameters such as WATER, NUTRIENTS, and biomass removal may have significant effects on community plant strategies indicated by the plant traits’ associations and trade-offs (ROOT GROWTH, LES, and SIZE AXIS) operating as stressors or plant soil resources (e.g., nutrient availability) (Grime 1998). For instance, ROOT GROWTH has been related to the acquisition of soil resources such as nutrients or water; therefore, variation of these parameters is expected to affect root growth (Prieto et al*.* 2015). However, here, we expect that high nutrient availability might limit root growth, as has been shown in similar systems where coastal nutrient enrichment drives an increase in above-ground biomass (e.g., leaves and stems) but a decrease of belowground biomass of bank-stabilizing roots of coastal salt marsh vegetation (Deegan et al. 2012)

In contrast, plant functional traits indicating root growth might respond positively under disturbance events such as biomass removal, as belowground biomass allocation has frequently been found in grasslands under extensive grazing management in relatively wet sites (Piñeiro et al*.* 2010). Alternatively, root growth stimulation might be expected under the influence of stressors such as seawater inundation, in order to improve their anchoring function in environments such as coastal saltmarshes (Nyman et al. 2006). No responses were expected from WATER to the other model variables (exogenous variable).

Here, we expected that reduction of the community aboveground biomass and canopy height (SIZE AXIS), driven by biomass removal and by the water gradient, might stimulate a resource acquisition strategy indicated by ROOT GROWTH (Piñeiro et al. 2010 and Bakker et al. 1993) and higher values of LNC and SLA (LES) (Lienin & Kleyer 2011). Therefore, we expected that higher values of plant traits pertaining to the SIZE AXIS (i.e., high community aboveground dry biomass weight and canopy height) may show a negative relation to several plant traits of the leaf economics spectrum (high SLA and LNC values, low LDMC values) and plant traits indicating ROOT GROWTH (high RSL, RNC, and low RDMC). The opposite relationships are expected on pure temperate salt marshes or alpine grasslands where the variation of abiotic parameters such as salinity/nutrients and altitude/nutrients respectively may determine a positive association between above-ground biomass and SLA values (Minden and Kleyer 2015, Lavorel and Grigulis 2012). However, here, we expect that elimination of highly competitive species (i.e., *Phragmites australis*) (higher biomass and canopy height) might drive the occurrence of vegetation displaying higher values of traits indicating a resource-acquisition strategy (SLA or/and RSL).

##### EP and ES trade-offs in response to plant functional traits

Bundles are set of ecosystem services that appear together repeatedly. The association can rise from common underpinning processes or as a response to common pressures (Mouchet et al. 2014). In general, a positive effect of above-ground biomass on belowground organic carbon stocks is expected (hereafter carbon stocks bundle), as larger plants are expected to shed more biomass in the form of leaf and woody litter in the ground and thus contribute directly to C accumulation in the standing litter and in the soil organic content (Conti & Díaz 2013; Doblas Miranda et al. 2013). It is expected that forage quality may indicate the economic benefit obtained by farmers (forage sales). Both parameters are associated with forage production for meat and dairy products; therefore, we call this group of parameters forage production bundle. Particularly in this system where mono-specific vegetation in non-disturbed patches are represented by very common dominant species, i.e., *Phragmites australis* or *Elymus athericu*s, it is expected that higher plant species’ richness positively affects endangered plant species’ occurrence (Joyce 2014); therefore, we call this group of variables plant nature conservation value bundle.

Biotic ecosystem properties associated with the carbon stock bundle (aboveground biomass and soil organic carbon) are expected to respond positively to plant traits explaining the size axis, i.e., higher aboveground dry weight, taller plants, higher leaf area and seed number (Conti and Díaz 2013; Díaz et al. 2004). Here, plant communities with these traits’ values are expected to be associated with plant mono-specific, undisturbed, and nutrient-rich plots, inhabited by, e.g., *Phragmites australis* or *Elymus athericus* (Esselink et al. 2000; Joyce 2014). However, here, contrary to what may be expected in other grassland systems (Lavorel & Grigulis 2012), we expected that higher levels of carbon stocks would be in trade-off with forage quality, and forage sale (forage production bundle) unused plots are expected to have higher standing biomass and higher SOC content (Conti and Díaz 2013, Lavorel & Grigulis 2012) (unused plots mean here plots non-disturbed by grazing or mowing, here inhabited by less relevant species for forage *Phragmites australis* or *Elymus athericus*)*.* Contrary to what may be expected in other systems (Tilman et al.  1996; Kumar 2011), here, we also hypothesized that higher carbon stock values would be in trade-off with the plants’ nature conservation value bundle (plant species richness and endangered plants occurrence), due to the expected association of higher above-ground biomass production with mono-specific species vegetation (Esselink et al*.* 2000). Contrary to what would be expected under niche complementarity conditions (Tilman 1997), in this system, with a high availability of soil resources (Esselink et al. 2000), we expect a positive selection effects on dominant species, with high resource consumption, compared to other species, resulting in high aboveground productivity and reduction of species richness (Joyce 2014). We expected therefore that this trade-off (i.e., non-disturbed vs. disturbed vegetation) would be explained by the variation of the size axis (i.e., canopy height, above-ground dry weight).

We expected that components of both the forage production bundle and the plant nature conservation value bundle would respond positively to biomass removal and therefore the variation of the size axis might show an initial association of these two bundles. However, by contrast, both plant nature conservation and forage production bundles may respond to traits’ variation on LES. It is expected that plant traits’ variation on the LES may be effective as a marker to differentiate between endangered plant species and forage sales. We hypothesized that traits’ variation on the LES might explain forage sales due to its relationship to foraging intensity (Lavorel & Grigulis 2012; Lamarque et al*.* 2014); thus, negative effects on endangered plant species are expected. High values of SLA and LNC (and low values of LDMC) may be good predictors of highly intensified fields (Lavorel and Grigulis 2012; Lamarque et al. 2014) and therefore higher sales associated with forage production.

Both LES and ROOT GROWTH were expected to be associated with higher plant species’ richness; conversely SIZE AXIS is expected to be associated with low plant species’ richness.

## Results

Below, we summarize main deviations from previous hypothesized initial model and present findings for (1) plant functional traits’ associations and trade-offs, (2) traits’ responses to environmental gradients, and (3) trade-offs between EP and ES in respond to plant functional traits.

Two of the six initially hypothesized latent variables were not retained in the model (ROOT GROWTH and LES) because several indicators (observed variables) from ROOT GROWTH and LES latent variables were not significant. After modification of these two latent variables (see modifications below), all the resultant five latent variables of the final model (Fig. 3) were significant based on quality measures (composite reliability coefficients (> 0.7) and average variances extracted (AVE) (> 0.5) (see SM 3 (Model Quality Measures)). Six paths were excluded because they were not significant (*p* > 0.05) (compare Figs. 2 and 3). Additionally, one direct pathway between PLANT GROWTH (i.e., co-variation of SRL and SSL indicating both rapid root and stem growth (Reich 2014; Freschet et al. 2010)) and endangered plants was added to improve the model performance, following theoretical justification (i.e., as plant communities with higher SRL and SSL might be associated with a resource acquisition strategy (Paula & Pausas 2011) or a vegetative strategy initiated by competition for survival (Moles & Westoby 2006). These strategies can be here triggered by a lower level of soil nutrients and disturbance event (e.g., mowing or grazing) characteristic from extensive wet grasslands richer in rarer plants (Esselink et al. 2000; Joyce 2014; Bakker et al. 1993).

The model showed a moderate explanatory power to predict two final services: forage sales, *R*^2^ 0.57, and endangered plant species, *R*^2^ 0.52.

### Plant functional traits’ associations and trade-offs

Both RNC and RDMC were not significant and therefore were removed from the initially hypothesized ROOT GROWTH. By contrast, specific root length (SRL) was kept in the model. Additionally, stem-specific length (SSL) and stem nitrogen content (SNC) were tested together with SRL, following empirical results of a coordinated plant economics spectrum found in certain systems (Freschet et al. 2010; Reich 2014). However, only SRL and SSL were retained in the model. The resulting significant latent variable was named PLANT GROWTH (i.e., co-variation of SRL and SSL indicating a rapid growth of both roots and stems in line with previous works showing co-variation between below- and aboveground plant traits (Freschet et al*.* 2010; Reich 2014)) (see latent variables’ consistency in SM 3). The leaf economics spectrum latent variable (LES) was modified because both LNC and LDMC indicators were not significant and therefore not retained in the model; only SLA was kept in the model due to its higher explanatory power. The SIZE AXIS latent variable was significant (SM 3)

### Traits’ responses to environmental gradients

Several expected direct paths from NUTRIENTS to plant traits were removed, and only the direct negative effect from NUTRIENTS to PLANT GROWTH was retained. The expected direct effect from WATER to PLANT GROWTH was removed, since on the contrary, a total negative effect was found (−0.31) due to the negative effect of WATER on biomass removal. The direct positive path from biomass removal to LES was not significant; however, this effect was confirmed by the total positive effect (0.41). The rest of the expected direct paths were correctly predicted (see Figs. 2 and 3). Table 3 shows total, indirect, and direct effects between variables (regressions’ beta path coefficients).

**Table 3:** Total (TE), indirect (IE), and direct effects (DE) between abiotic ecosystem properties, biotic ecosystem properties, land use, and values of final ecosystem services

		Abiotic EP			Land use			Plant traits											
		NUTRIENTS, *R*^2^ 0.39			Biomass removal			PLANT GROWTH, *R*^2^ 0.74			SLA, *R*^2^ 0.67			SIZE AXIS, *R*^2^ 0.43					
		TE	IE	DE	TE	IE	DE	TE	IE	DE	TE	IE	DE	TE	IE	DE			
Abiotic EP	WATER	0.62		0.62	−0.57		−0.57	−0.31	−0.31		−0.64	−0.01	−0.63	−0.01	0.44	−0.45			
Abiotic EP	NUTRIENTS							−0.19		−0.19									
Land use	Biomass removal							0.82	0.47	0.35	0.41	0.41		−0.80		−0.80			
Plant traits	SIZE AXIS							−0.58		−0.58	−0.51		−0.51						
		Biotic EP												Final ecosystem services					
		SOC, *R*^2^0.22			AGB, *R*^2^ 0.57			FORAGE QUALITY, *R*^2^ 0.36			Plants, *R*^2^0.55			Endan. plants, *R*^2^ 0.52			Sales, *R*^2^ 0.57		
		TE	IE	DE	TE	IE	DE	TE	IE	DE	TE	IE	DE	TE	IE	DE	TE	IE	DE
Abiotic EP	WATER	−0.29	-0.29		0.00	0.00		0.00	0.00		−0.23	−0.23		0.23	0.23		−0.37	−0.37	
Abiotic EP	NUTRIENTS	−0.17	-0.17								−0.14	−0.14		−0.12	−0.12				
Land use	Biomass removal	0.23	0.23		−0.63	−0.63		0.38	0.38		0.61	0.61		0.23	0.23		0.35	0.35	
Plant traits	PLANT GROWTH	0.90		0.90							0.74		0.74	0.62		0.62			
Plant traits	SLA		0.12											−0.66		−0.66	0.59		0.59
Plant traits	SIZE AXIS	0.12			0.79		0.79	−0.47		−0.47	−0.43		−0.43	−0.06		−0.06	−0.44	−0.44	
Biotic EP	AGB	0.80		0.80				−0.6	−0.6								−0.17	−0.17	
Biotic EP	FORAGE QUALITY																0.29		0.29
Biotic EP	Plants													0.83		0.83			

*N* 44. All path coefficients are significant at *p* < 0.05. The effect values represent regression beta path coefficients *β* from the partial least squares structural equation model results

*R*^*2*^ R-square values obtained by the predicted variable in the PLS-SEM

### Trade-offs between EP and ES in respond to plant functional traits’ variation

Endangered plants’ occurrence and plant species’ richness (plant nature conservation value bundle) were moderately well explained by the model (*R*^2^ 0.52 and *R*^2^ 0.55 respectively) as were forage quality and forage sales (forage production bundle) (*R*^2^ 0.36 and *R*^2^ 0.57 respectively, Table 3). SOC and AGB were slightly and substantially explained by the model (*R*^2^ 0.22 and *R*^2^ 0.62, respectively, Table 3).

The hypothesized trade-offs and associations between properties and services were confirmed by the indirect and direct effects from plant functional traits’ variation. However, some slight deviations were found.

The results revealed an additional trade-off between plant functional traits: High values of SLA were negatively associated with endangered plant species, and PLANT GROWTH was positively associated with endangered plant species (Fig. 3)

A relatively small indirect effect from SIZE AXIS variation on SOC was found (0.12) (Table 3). However, variation of SIZE AXIS showed two significant, indirect opposite paths on SOC (see Fig. 3 and Table 3). Increments on the SIZE AXIS showed a positive effect on SOC via increasing AGB. In contrast, increments of SIZE AXIS showed an alternative, negative effect on SOC via decreasing values of PLANT GROWTH. Subsequently biomass removal showed opposite, indirect effects on SOC.

PLANT GROWTH showed an indirect, positive effect on the habitat value to conserve endangered plant species and a direct positive effect on the same variable (see Table 3).

## Discussion

This research paper aimed at disentangling (1) associations and trade-offs between plant functional traits within a particular context of North-West European coastal grassland vegetation, including plant strategies associated with plant growth, leaf economics spectrum, or size axis. Do we find negative associations between traits? Do some traits co-vary? How are these relations coordinated through the whole plant, including below- and aboveground biomass?; (2) how environmental factors and land use predict trait relationships; and (3) to what extend trait associations and trade-offs can predict ecosystem properties, e.g., carbon stocks or forage quality, and final ecosystem services, i.e., habitat value to conserve endangered species or sales of forage-based products, i.e., meat and dairy products.

### Coordination of plant functional traits

#### Coordination of plant growth, LES, and SIZE AXIS

In line with what has been shown in other North-West European grasslands (Lienin & Kleyer 2011), variation of plant traits’ values associated with a size axis (canopy height, above-ground dry weight, and leaf area) co-varied negatively with traits expressing the LES, such as SLA. Here, we also found a strong association between the size axis and the vegetation reproductive abilities (seed number). In addition, we also found a negative association with high values of plant functional traits associated with a plant-growth and soil-resource acquisition strategy (i.e., SRL and SSL). Thus, taller vegetation showed higher biomass values (size axis), but, in contrast, lower values for several traits explaining an extreme of a plant economics spectrum or leaf economics spectrum, i.e., SRL, SSL, and SLA. Empirical results from Díaz et al. (2004) suggested the existence of at least these two axes in plant functional traits’ variation. However, a general co-variation between these two axes has not been proven, at least on a global scale. On the contrary, an orthogonal relationship between these two axes was found suggesting independency between these two gradients, indicating different but complementary plant strategies Díaz et al. (2004).

Contrary to our results, a positive association between high values of traits belonging to the size axis and high values of traits belonging to a resource-acquisition plant strategy was reported in other systems, such as European alpine grasslands (Lavorel & Grigulis 2012) or Mediterranean woody vegetation (Riva et al*.* 2016). A positive association between high values for traits such as canopy height (size axis) and high values of LNC was also reported in the alpine system. This was explained by the fact that altitude may gradually act as a strong negative filter for plant height and a resource-acquisition strategy (higher LNC values) because of resource limitations, such as water or nutrient availability. These empirical results showed how vegetation is situated in a co-variation of a strong acquisition vs. conservation gradient and a size axis gradient. However, in our system, as is explained in detail below, neither nutrients nor groundwater gradient played an important direct role as environmental filters when compared to disturbance, due to the fact that both nutrients and water were not scarce. Therefore, here, the variation in the plant functional traits was mainly related to a major axis associated with a disturbance gradient.

Our results showed an initial major co-variation between leaf, root, and stem traits, suggesting a potential plant economics spectrum coordination as shown for sub-arctic or semi-arid environments in which a limitation of resources occurred along an environmental gradient (Freschet et al. 2010; Riva et al. 2016).

#### Fine roots and fine stem growth (plant growth)

High values of RDMC or SDMC are often situated at one extreme of the trade-off between resource acquisition and resource conservation vegetation strategies (Riva et al. 2016). Higher values of these traits indicate a vegetation with higher lignin content for roots and stems and a development of a resource conservation strategy in response to plant resource limitation (Lopez-Iglesias et al. 2014). At the other extreme of the gradient, higher values of SRL, SSL, RNC, and SNC might indicate the development of a resource-acquisition strategy under certain environmental conditions. For example, plant seedlings might develop long fine roots (i.e., high SRL) for soil exploration and improvement of water uptake efficiency under drought conditions in Mediterranean systems as a survival strategy (Paula & Pausas 2011). However, here, neither RDMC, SDMC, nor RNC and SNC were in significant trade-off with SRL or SSL. This result might indicate that plants here did not need to reinforce a resource conservation strategy, probably due to the fact that groundwater and soil nutrients were not scarce enough (Lavorel & Grigulis 2012).

#### Leaf economics

Contrary to expectations, several variables explaining the leaf economics spectrum, i.e., leaf nitrogen content (LNC) and leaf dry matter content (LDMC) (Wright et al. 2004; Gardarin et al. 2014), were not significant and therefore did not explain the expected latent variable LES (leaf economics spectrum). This might be related to the fact that the system was not nutrient limited (see Table 2), contrary to what has been shown in European alpine grasslands (Lavorel & Grigulis 2012) or Mediterranean forests (Riva et al. 2016). Therefore, these plant traits did not express a significant variation because there was no need for a resource conservation strategy according to C-S-R Grime’s scheme (1998) for major plant strategies. However, SLA was kept in the model as a single variable because it showed a relatively high explanatory power, probably due to the fact that (1) plants from salt marshes showed a low SLA due to the occurrence of plants with succulent leaves and (2) plants from intensive grassland showed high values for the same parameter (Table 2). In other studies, SLA has been shown to have a higher explanatory power than LDMC (Hodgson et al. 2011).

### Environment-traits’ effects

We did not find a significant positive effect of soil nutrient availability on plant traits, probably due to the strong effect of biomass removal in the plant traits’ responses (Lienin and Kleyer 2011) and probably also due to the fact that nutrient availability was relatively high in the system. Despite the expectation of positive effects of soil nutrient availability on the traits such as SLA and aboveground dry weights (Garnier et al. 2007), here, this effect was masked by the effect of environmental parameters such as the water gradient (positive co-variation of salinity and groundwater) and disturbances such as biomass removal. In contrast, increasing values of soil nutrients showed a negative effect on plant growth, i.e., specific root length and specific stem length. Contrary to expectations from a plant economics spectrum perspective (Freschet et al. 2010), high nutrient availability may limit a resource-acquisition strategy in highly productive systems. This effect has been found in empirical studies in North American temperate coastal grasslands, where increasing values of soil eutrophication have been shown to significantly modify the root to shoot ratio, with subsequent negative effect on sediment retention (Turner et al. 2009; Deegan et al. 2012).

Contrarily to expectations (Nyman et al. 2006), the water gradient showed neither a significant direct positive effect nor a total positive effect on the plant growth strategy. A total insignificant effect of the water gradient on the plant growth strategy was probably due to two confirmed but opposite mechanisms of plant growth stimulation (Fig. 3): (1) positive effect by the water gradient following Nyman et al. (2006) and Bakker et al. (1993); and (2) indirectly negative effect by water gradient via segregation of biomass removal landwards. The strong effect of biomass removal on plant traits’ variation probably masked the effects of the water gradient.

### Trade-offs between ecosystem properties and ecosystem services in response to plant functional traits’ variation

#### Decoupling plant growth and leaf economics spectrum

Higher values of plant traits indicating plant growth, such as SRL and SSL, might be associated with a resource acquisition strategy (Paula & Pausas 2011) or a vegetative strategy initiated by competition for survival (Moles & Westoby 2006). Contrary to expectations on a whole-plant economics spectrum (Freschet et al. 2010), here, a plant-growth strategy was decoupled from components of the LES (SLA). On the one hand, SLA negatively explained habitat value to conserve endangered plant species and positively explained forage sales; on the other hand, high values of plant growth positively explained habitat value to conserve endangered plant species. These findings indicated two different plant strategies whose operation depended on land use variation. (1) Plant growth strategy (fine roots and fine stems growth) was triggered by biomass removal under extensive management on grasslands, generating a higher plant richness and a subsequent competition for resources and survival (i.e., light and soil nutrient uptake) (Freschet et al. 2010; Prieto et al. 2015). By contrast, (2) higher SLA values, despite being also triggered by biomass removal, are probably here associated with a higher intensification of grassland patches (i.e., here indicated by higher levels of soil nutrients availability in combination with biomass removal) which might indicate a lower competition for soil resources (Lavorel & Grigulis 2012; Kleyer & Minden 2015). This may imply that plants do not allocate resources to roots and stems, and they are therefore available to be allocated to leaves (Minden & Kleyer 2014).

#### Traits explaining SOC in respond to land-use variation

A total negative effect of size axis reduction on soil organic carbon due to biomass removal was not obvious in this system. Two opposite mechanisms explained indirect effects of biomass removal on SOC. On the one hand, an overall negative effect was found due to the fact that removal of aboveground biomass might reduce both litter accumulation and an overall belowground productivity (Conti & Díaz 2013; Doblas Miranda et al. 2013). Our results show that higher values of aboveground biomass had a positive effect on soil organic carbon. However, there was a secondary and opposite path on soil organic carbon accumulation associated with fine root growth. We found a positive effect of plant growth (RSL and SSL) on soil organic carbon, due to the fact that biomass removal may trigger belowground productivity of fine roots and subsequent soil organic carbon accumulation (Yu & Chmura 2009; Piñeiro et al. 2010). We suggest that a potential trade-off between these two opposite paths masks the total indirect effect of biomass removal on soil organic carbon, so that this is not visible in the resulting model.

#### Forage sale responses to SLA and forage quality

Community-based forage quality (FORAGE QUALITY) did show a slightly positive effect on forage sales. Contrarily, SLA did show a stronger significant effect on forage sales. This fact highlighted the strong explanatory power of a single trait, such as SLA, which was in line with that found by Gardarin et al. (2014). SLA was a better predictor of forage sales than forage quality here, due to the fact that salt marsh vegetation does show relatively high values of forage quality, but relatively low values of SLA. However, we have to caveat that apart from the forage consumed by cattle directly on the field, additional forms of supplementary feeding might have happened though were not accounted for in this study and therefore it may have increased the model uncertainty.

## Conclusions

Under the threats of environmental change and strong land use modifications, an accurate knowledge about the response of biodiversity and ecosystem properties to environmental and land use gradients and their effects on ecosystem services is crucial (Duncan et al. 2015, Bennett et al. 2009). Trade-offs and synergies between ecosystem services can be initially identified spatially (Raudsepp-Hearne et al. 2010). However, these associations are highly dependent on drivers of change and processes affecting ecosystems (Eigenbrod et al. 2010) and, to a large extent, vegetation (DeBello et al. 2010), making these associations highly variable in time and space, due to the variable nature of environmental factors (de Deyn et al. 2008) and socio-ecological relationships (García-Llorente et al. 2015, Santos-Martín et al. 2013). Therefore, land use optimization models aiming at determining the provision of ecosystem services at landscape level should include at first step a broad spectrum of biophysical and socio-ecological direct and indirect causal relationships which predict final services (Cebrián-Piqueras et al. [Bibr CR6][Bibr CR7]).

Trait-based approaches like the one presented here can serve as first step to further development of spatially explicit models predicting distribution of ecosystem services at landscape scale as shown by Lavorel et al. (2011) and Lamarque et al. (2014). Correspondingly, comparison and cross-validations between regions and gradients can be crucial for a better understanding of plant communities and plant strategy responses to environmental and land use changes (Díaz et al. 2004). Besides, considering a broad spectrum of plant functional traits might allow understanding and predicting the mechanism that control plant strategies (Kleyer et al. 2018) in response to environment variation (Freschet et al. 2010).

The approach here presented showed significant interactions between environmental gradients, plant traits, ecosystem properties, and services that were explained by a structural equation model*.* A dichotomy between non-disturbed and disturbed vegetation was shown to be responsible for a major trade-off between aboveground carbon stocks and both plant nature conservation value and forage production (i.e., habitat value to conserve endangered plants and plant richness and forage quality and forage sales respectively). This trade-off was explained by a negative co-variation between plant traits associated with the size axis and with traits pertaining to the plant economics spectrum. However, a secondary trade-off related to detailed variation between extensive vs. intensive management, explained a trade-off between nature conservation value and forage production (i.e., habitat value to conserve endangered plant species and forage sales, respectively). This ecosystem services trade-off was explained by a trade-off between a plant strategy associated with plant growth (both roots and stems) and a plant strategy associated with leaf resource acquisition (higher SLA values). This result suggests the existence of allometric patterns that operate independently between several plant parts in response to environmental gradients, as has been suggested in other systems (Fortunel et al. 2012). These results are contrary to the generalization of a co-variation of leaf, stem, and root traits indicated on a plant economics spectrum, which is expected to function under other environmental conditions (Freschet et al. 2010). Contrary to the initial expectations, variation of land use intensity did not significantly affect the soil organic carbon stocks due to two opposite, alternative mechanisms explained by variation of the size axis and plant growth.

Land use intensity, quantified here as biomass removal, was found to be the main direct environmental driver of plant trait responsiveness on these temperate coastal grasslands, which, on the other hand, was strongly affected by the co-variation of groundwater and salinity levels. Contrary to the plant or leaf economics, our results suggest that the trade-offs and synergies between plant functional traits found here are not explained by a resource acquisition vs. resource conservation trade-off. Here, the variation of plant functional traits in respond to disturbance may express a trade-off on a gradient of well-established competitors’ species (persistence) vs. competition for establishment (growth) (Grime 1998; Minden & Kleyer 2014). This gradient seems not to respond to soil-nutrient availability, in contrast to biomass removal. Abundance of soil-available nutrients might, however, explain how later stages of vegetation succession may be associated with competitor mono-specific vegetation, such as *Phragmites australis* in fresh and brackish water and *Elymus athericus* in high-salt marsh vegetation.

These results indicate that plant community assemblies and associated specific key plant traits can well explain the variation of determinant environmental parameters and their effects on ecosystem properties and services. Empirical approaches, as used here, may help to optimize management strategies under the threats of environmental change and strong land-use pressure. For instance, from a nature conservation perspective, as shown by these findings and in line with other studies, maintenance of plant species richness and rarer species in coastal wet grasslands (salt, fresh, or brackish) might require conservation management strategies such as extensive grazing or mowing (Esselink et al. 2000; Joyce 2014; Bakker et al. 1993), with subsequent probable positive effects on belowground biomass production and associated carbon stocks. Additionally, combination of previous measures with a reduction of anthropogenic sources of soil nutrients, i.e., excess of agricultural fertilization, might limit the spread of highly competitive mono-specific vegetation, facilitating richer plant communities with higher capacity to explore soil resources (growth of fine roots) and therefore contributing to maintenance of soil carbon stocks. In contrast, from an intensive forage production perspective, these results reveal the importance of leave traits (SLA), as opposed to stem and root traits (SSL or SRL) (plant growth), to predict the capacity of coastal vegetation to provide agricultural benefits from cattle grazing and mowing.

We highlight the applicability of functional traits to predict environmental changes and effects on ecosystem properties and services. Although here not all previously expected plant traits responded to the environmental gradients (e.g., RDMC or RNC), this does not imply that they might not be functional in other gradients as it has been shown in other systems (Lavorel & Grigulis 2012; Paula & Pausas 2011; Prieto et al. 2015; Freschet et al. 2010). Therefore, considerations should be taken in order to generalize the results here obtained for other systems due to the strong place-based nature of this work. We argue that more study cases are needed to explore the applicability of the effect-response framework in relation to the prediction of ecosystem services variation under several environmental gradients (Hevia et al. 2017; Kleyer et al 2018). Besides, consideration of different parameters of functional diversity, i.e., functional richness, evenness, or divergence, should be considered to specifically test the role of trait diversity in controlling ecosystem functions and services (Wen et al. 2019). Our study confirmed the potential applicability of plant trait values retrieved from regional level data sets in a specific site context; however, further studies should be conducted to compare the efficacy of both site- and regional-based data sets to explain responses and effects of vegetation (Cordlandwehr et al. 2013). Finally, we argue that to address scientific and management questions about the provision of multiple services, progress is desirable for understanding how functional trade-offs and synergies within biodiversity scale up to interactions between ecosystem services. Ecosystem service management within the context of global change, land use change, or ecological restoration remains a major challenge, but trait-based understanding opens new avenues towards more integrated approaches (Lavorel 2013). Nevertheless, we highlight that further research is needed to explore the potential of trait-based approaches to explain landscape interaction of ES (Lavorel et al. 2011).

## Supplementary information

ESM 1 (DOCX 383 kb)
