# Supplementary material for: Digging into the roots: understanding direct and indirect drivers of ecosystem service trade-offs in coastal grasslands via plant functional traits
Source: Environ Monit Assess. 2021 May 14;193(Suppl 1):271. doi: 10.1007/s10661-020-08817-x (PMC8121717; doi:10.1007/s10661-020-08817-x)
Supplement: Supplementary file 1 — (DOCX 383 kb) [file 10661_2020_8817_MOESM1_ESM.docx]

# Supplementary Material (SM)

# SM 1: Trait collection and measurement

Traits were collected from healthy, fully grown plant individuals, usually at the phenological stage “seeds are ripe but not yet shed”. In order to assess allocation strategies, all traits used in this study were taken from the same individual.

In each study region (Table 1), we recorded plant species’ frequencies in 1 m² plots. In each plot, we selected one or two plant individuals. These were unitary plants (e.g. annuals) or ramets in case of clonal species. Altogether, 8 to 10 individuals were selected per species from as many plots as possible. To protect local populations of rarer species, we collected only one or two individuals of these species.

A square of size 30x30cm with the focal ramet in its center was cleared of all other plants. The inflorescence with seeds was clipped and stored in a paper bag. The stem with leaves was clipped approx. 4 cm above the soil surface, wrapped in wet paper, and put in a sealed plastic bag. The remaining stem fragment was marked with a colored tape near the soil surface. Throughout further processing, careful labelling of all organ-specific samples was essential to assign them to a given individual and its plot.

The cleared square was then excavated to a depth of 30 cm and the soil core was carefully gathered into a 30 l plastic bag (PE trash liners with handle). All fresh plant material was transported to the laboratory in cooler boxes to prevent weight loss. The shoot material was immediately stored in a freezer. The soil was carefully rinsed off the roots. The roots and rhizomes of the focal individual were separated from the roots of other plants. We are aware of the limitations of this approach when roots extend over larger distances than 30x30x30cm, either vertically or laterally. Fine roots are likely to be undersampled and live roots are sometimes hard to separate from dead roots (Poorter *et al.* 2012). We also separated the leaves from the stems, selected and immediately froze 2 leaves and 2-4 stem, root segments for detailed measurement and oven-dried the remaining organ tissue (72h at 70°C).

For detailed measurements, the leaves as well as stem, root segments were defrosted and rehydrated in water (approx. 10 min.). The petiole of each leaf was separated from the blade, rubbed dry and scanned with a flatbed scanner. Before scanning, the fresh weight of the leaves and petioles was determined. Leaf area was derived from the scans with the software Image J (https://imagej.nih.gov/ij/index.html). Finally, the leaves and petioles were oven-dried (72h at 70°C), weighed and added to the other leaf tissue of this individual. Until further processing, the dried material was stored in desiccators.

For each individual leaf, we calculated leaf area [LA; mm²], specific leaf area (SLA; leaf area [mm] / leaf weight [mg]), and leaf dry matter content (LDMC, leaf dry weight [mg] / leaf fresh weight [g]). Values were averaged to produce a mean LA, SLA, and LDMC per individual or ramet. We also measured leaf dry biomass of the individual or ramet (total leaf dry weight [g]; LDW).

Density of stem and root segments was analysed with the volume replacement method using a volumetric flask (“Pycnometer”). In species such as *Phragmites australis*, the central hollow of the stem was considered to be an air space and not included in the volume, but smaller xylem vessels were included. After defrosting, we rehydrated the segments and measured the length of the segments as precisely as possible. Afterwards, the segment fresh weight was determined. After measuring the weight of the water-filled flask, the segment was immersed in the flask and the weight measurement repeated. The difference between both measurements gave the volume of the segment. Finally, the dry weight of the segment was measured and added to the dry weight of the remaining stem and root material. From these measurements, we calculated stem and root specific length (SSL, SRL; segment length [mm] / dry weight [mg]). We also used stem, root or rhizome biomass of the individual or ramet (SDW and RDW ; total stem or root dry weight [g]). For species collected with seeds we counted their number (SN).

The dried leaf, stem and root samples of each individual were ground to a fine powder using a Retsch mixer mill mm400. These samples were analysed with Near-Infrared Spectroscopy (NIRS, Bruker MPA Spectrometer). Additionally, nitrogen concentration per dry mass were analysed with a C/N Analyser for approx. 20% of the samples. These measurements were used for the calibration of the NIRS spectra. N concentrations of all samples were predicted with partial least squares regressions (Bruker OPUS Software), resulting nitrogen concentration of leaves, stems, roots, rhizomes, and seeds (LNC, SNC, RNC)

**SM 2:** Locations where the trait-plants where collected: 1) Zeeland region in The Netherlands; 2) Coastal lowlands of Norwest Germany including the “National Park Wadden Sea” and several coastal barrier islands of the North Sea coast; 3) “Nationalpark Mols Bjerge” located in Danish Baltic Sea Coast; 4) National Park “Vorpommersche Boddenlandschaft” in the German Baltic Sea coast. Trait-plants where collected from four main vegetation units occurring in all four regions (i.e. saltmarshes, extensively managed wet grasslands, intensively managed grasslands and reeds).


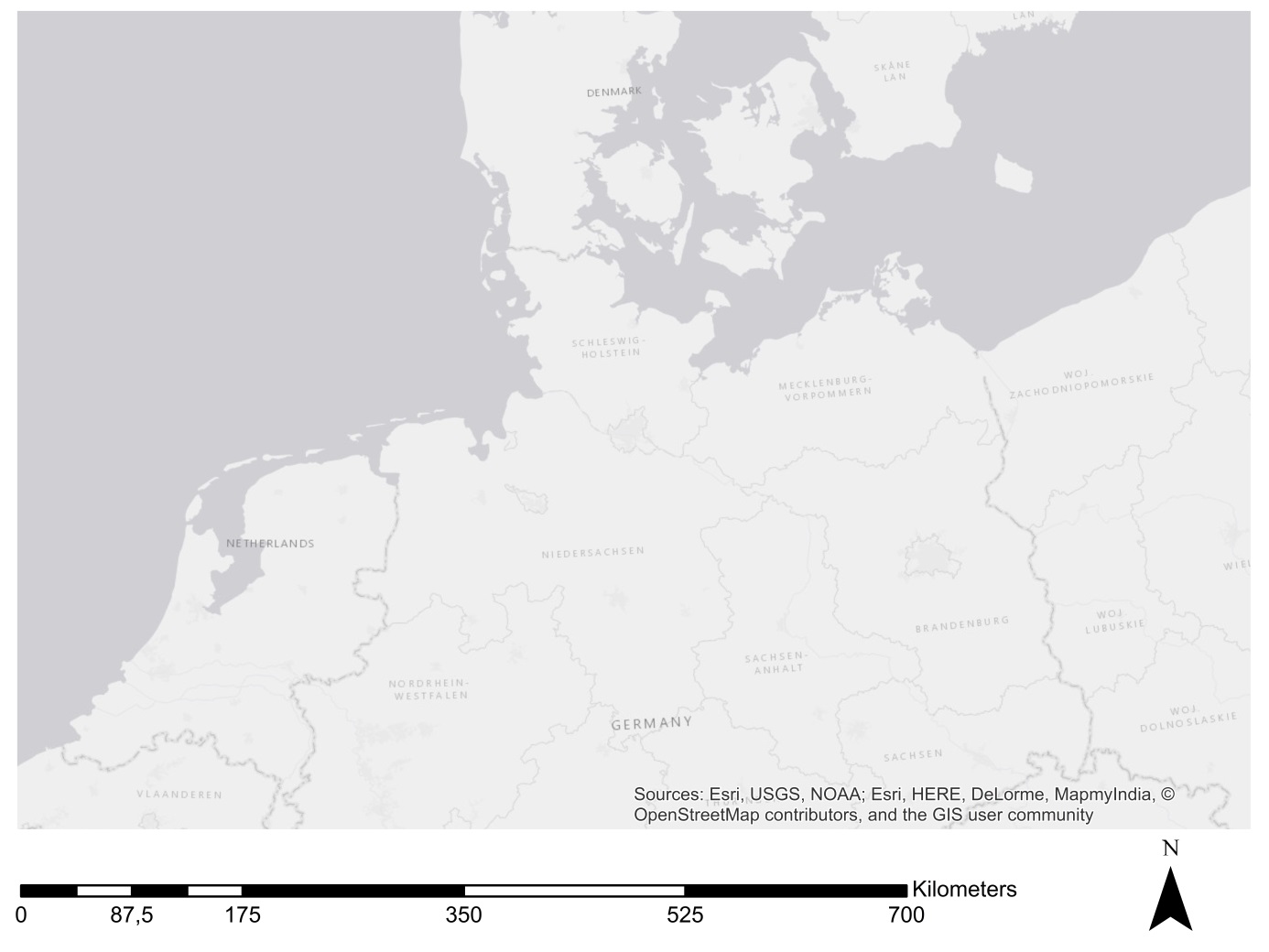


4

2

1

3

**SM 3:** Final model quality measures for latent variables. Abbreviations; WATER: Co-variation of salinity and groundwater levels: Nuts: Soil available nutrients.

| Latent variable coefficients | |  |  |  |  |
| --- | --- | --- | --- | --- | --- |
|  | PLANT GROWTH | SIZE AXIS | WATER | FORAGE  QUALITY | Nuts. |
| Composite reliability | 0.90 | 0.98 | 0.87 | 0.86 | 0.77 |
| Average variances extracted (AVE) | 0.83 | 0.94 | 0.77 | 0.76 | 0.54 |
| R-Square | 0,74 | 0,43 |  | 0,36 | 0,39 |
